# Long-term results of transpedicle body augmenter in treating burst fractures

**DOI:** 10.4103/0019-5413.37001

**Published:** 2007

**Authors:** Allen Li, Jung-Kuei Chen, Kung-Chia Li, Ching-Hsiang Hsieh

**Affiliations:** Department of Biology, Johns Hopkins University, Baltimore, MD 21218, USA; * Department of Orthopaedic Surgery, Armed Forces Taoyuan General Hospital, Taoyuan, Taiwan, ROC; ˆ Department of Orthopaedic Surgery, Chiayi Yang-Ming Hospital, Chiayi; Taiwan, ROC; # Institute and Faculty of Physical Therapy, National Yang-Ming University, Taipei, Taiwan, ROC

**Keywords:** Burst fractures, kyphosis, posterior instrumentation, spinal trauma, thoracolumbar injury, transpedicle body augmenter

## Abstract

**Background:**

Short-segment fixation alone to treat thoracolumbar burst fractures is common but it has a 20-50% incidence of implant failure and rekyphosis. A transpedicle body augmenter (TpBA) to reinforce the vertebral body via posterior approach has been reported to prevent implant failure and increase the clinical success rate in treating burst fracture. This article is to evaluate the longterm results of short-segment fixation with TpBA for treatment of thoracolumbar burst fractures.

**Materials and Methods:**

Patients included in the study had a single-level burst fracture involving T11-L2 and no distraction or rotation element with limited neurological deficit. Patients in the control group (n = 42) were treated with short-segment posterior instrumentation alone, whereas patients in the augmented group (n = 90) were treated with a titanium spacer designed for transpedicle body reconstruction. The followup was 48-101 months. The radiographic and clinical results were evaluated and compared by Student's t test and Fisher's exact test.

**Results:**

The blood loss, operation time and hospitalization were similar in both the groups. The immediate postoperative anterior vertebral restoration rate of the augmented group was similar to that of the control group (97.6% ± 2.4% *vs*. 96.6% ± 3.2%). The final anterior vertebral restoration was greater in the augmented group than in the control group (93.3% ± 3.4% *vs*. 62.5% ± 11.2%). Immediate postoperative kyphotic angles were not significantly different between the groups (3.0° ± 1.8° *vs*. 5.1° ± 2.3°). The final kyphotic angles were less in the augmented group than the control group (7.3° ± 3.5° *vs*. 20.1° ± 5.4°). The augmented group had less (*P* < 0.001) implant failure [0% (n=0) *vs*. 23.8% (n=10)] for the control group) and more patients (*P* < 0.001) with no pain or minimal or occasional pain (Grade P1 or P2) than the control group [90.0% (n=81) *vs*. 66.7% (n=28)]. All patients in the augmented group and 39 (92.8%) patients in the control group experienced neurological recovery to Frankel Grade E. Three patients in the control group had improvement to Frankel Grade D from Frankel Grade C, but later had deterioration to Frankel Grade C because of loosening and dislodgement of the implant.

**Conclusion:**

Posterior body reconstruction with TpBA can maintain kyphosis correction and vertebral restoration, prevent implant failure and lead to better clinical results.

Various methods were designed to treat thoracolumbar burst fractures. Nonoperative treatment including bed rest and mobilization with brace^1^–^3^ or surgical alternatives^4^–^8^ including posterior instrumentation or anterior plating have been tried and had their limitations.^5^,^9^–^11^ Posterior short-segment fixation is the most common and simple treatment.^4^–^6^,^8^ However, short-segment fixation alone led to a 20-50% incidence of implant failure and an increase in kyphosis in the long term.^5^,^10^,^12^ Methods including transpedicle bone grafts^13^ and polymethylmethacrylate^14^ have been used to prevent implant failure. However, transpedicle bone grafts have not prevented early failure of the implant^12^ and may lead to low anterior interbody fusion rates in the long term.^15^,^16^ The polymethylmethacrylate vertebroplasty was reported to prevent implant failure,^14^ but the longterm results are unknown, with possible cement complications.

A transpedicle body augmenter (TpBA) to reinforce the vertebral body via posterior approach has been reported to prevent implant failure and increase the clinical success rate in treating burst fracture and Kümmell's disease.^17^,^18^ The TpBA within the vertebral body, like cages in the disc space, was designed to provide internal mechanical support to maintain the structural height.^19^ The biomechanical advantages of transpedicle body augmentation in burst fractures has been documented in the porcine spine.^20^

The operation time and blood loss for posterior fixation and vertebral body reconstruction with the augmenter was similar to posterior fixation alone and the additional augmentation helped maintain the reduction of the vertebral body, leading to clinical success in the short term.^17^ However, the longterm results were not reported before. This study was done in order to further understand the longterm outcome of the TpBA in treating burst fracture with short-segment fixation.

## MATERIALS AND METHODS

We retrospectively reviewed 162 patients with thoracolumbar burst fractures who were treated with posterior short-segment fixation from January 1998 to June 2003. Patients included in the study had neurological function limited to Frankel Grades C, D or E.^21^ a single-level burst fracture (Type A3.3 trauma according to classification of Magerl *et al*.^22^) with more than 6 points graded by the load-sharing mechanism described by Gaines *et al*.^23^ and McCormack *et al*.^24^ (maximum of 9 points, comprising the comminution, displacement and kyphosis correction), limited involvement of T11-L2, no distraction or rotation component, no other major organ system or musculoskeletal injuries and a nonpathologic fracture. Because we excluded all cases with other organ trauma, the general conditions were stable in these cases. All the operations were done within 48 h after the patients were sent to our hospital and within seven days after fracture occurred. The augmented group received short-segment posterior fixation and reinforcement with the transpedicle body augmenter (Merries International Inc, Taipei, Taiwan), which is a titanium spacer with a bone ingrowth surface of different sizes (7 × 9 × 20 mm, 9 × 11 × 27 mm, 10 × 13 × 27 mm, etc).^9^ The control group was treated with short-segment posterior fixation alone. In the early stage of the study, more patients were treated with pedicle screws alone and in the later stage of the study, more patients were treated with the augmenter.

The clinical results were based on the latest followup as of May 31, 2007. The followup rate was 81.5%. Eight patients died of unrelated medical illnesses and 22 patients were lost to followup. These 30 patients were excluded from this retrospective study; 132 patients (90 in the augmented group, 42 in the control group) were included [Table 1]. The mean followups were similar for both groups, 68 months (range, 48-96 months) for the augmented group and 70 months (range, 50-101 months) for the control group. The mechanisms of injury included fall from height (62.1%) and road traffic accidents (37.9%).

**Table 1:** Patient demographics

Demographics	Group A (n = 90)	Group B (n = 42)
Age in years, mean (range)	58 (29-89)	57 (30-82)
Male to female ratio	37:53	18:24
Fracture location		
T11	18	8
T12	24	12
L1	29	13
L2	19	9
Load-sharing scores, mean (range) Neurological deficit (Frankel grade)	7.5 (6-9)	7.6 (6-9)
C	22	14
D	38	19
E	30	9

The preoperative evaluation protocol included anteroposterior (AP) and neutral lateral thoracolumbar radiographs, computed tomography (CT) scans and magnetic resonance imaging (MRI) scans to evaluate fracture sites and cord compression status. The pedicle size was measured on the CT or plain AP and lateral radiographs.

**Figure 1:**
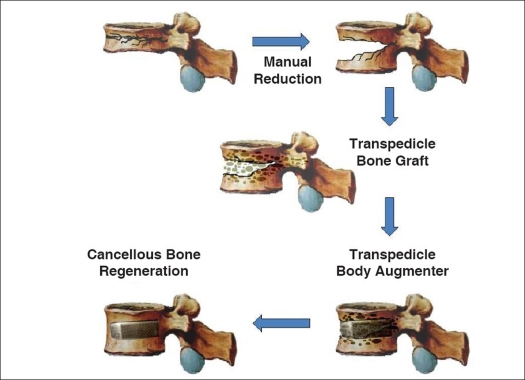
The flow chart shows the procedure for inserting the bone graft and augmenters

All the patients initially received manual reduction and short-segment fixation. Manual reduction was done as Li's previous reports.^17^,^18^ The acute burst fractures were usually reduced easily by the manual procedure, but left a substantial bony defect in the vertebral body. After manual reduction, pedicle screws were placed at the level above and below the fractured vertebrae (two levels, four screws) using the rod screw system (Reduction-Fixation Spinal Pedicle Screw System, Advanced Spine Technology Inc, Oakland, CA; Diapason, Stryker Corp, Allendale, NJ; and UP spine system, Titec Medical Co. Ltd, Taipei, Taiwan, Merries spine system, Merries International Inc, Taipei, Taiwan). Bilateral pedicle tunnels to the fractured vertebral body were made by an awl, followed by serial custom made trials (enlargers) to prepare for TpBA passage. The bony defect in the fractured vertebral body was filled through bilateral pedicle tunnels,^13^,^17^,^18^ with autologous bone graft mixed with calcium sulfate (OSTEOSET^®^, Wright Medical Technology, Arlington, TN) if the autograft from the posterior iliac bone was insufficient. Then the augmenter was inserted into the vertebral body through the pedicle tunnel and finally, bone graft was used to fill the pedicle tunnel space [Figure 1]. Patients wore a thoracolumbar brace for three months. After discharge, patients were followed up regularly. Operation time, blood loss, hospitalization and complications were documented.

Serial radiographs (supine AP and lateral films centered on L1) were obtained regularly at immediate and 3 months after surgery. Flexion and extension radiographs were obtained after one year and at the final visit.

The sagittal plane kyphosis angle was measured as described by Kuklo *et al*.,^25^ from the superior end plate of the vertebral body above the fracture to the inferior end plate of the vertebral body below the fracture level. The predicted anterior vertebral body heights were estimated by the mean of the heights of the upper and lower adjacent segments. The kyphosis angle and anterior body heights were measured on neutral thoracolumbar radiographs before the operation, immediately after surgery and at the final followup. All digitization and measurements were done using EBM-viewer software (EBM Technologies Inc, Taipei, Taiwan) with an accuracy of ± 0.1 mm. Clinical results were assessed by the performance scale (Grades A-E) described by Frankel *et al.*^21^ and the pain scale (Grades P1-P5) described by Denis *et al*.^26^ The Denis pain scale vary from P1 as no pain; P2, occasional, minimal pain with no need for medication; P3, moderate pain, occasional medications, no interruption in work or activities of daily living; P4, moderate to severe pain, occasional absence from work, significant change in activities of daily living; and P5, constant severe pain requiring chronic medications.

Student's t test was used for statistical analysis of kyphotic deformity, kyphosis correction and anterior vertebral height. Fisher's exact test was used to analyze the instrument failure rate and pain scale data. The data are presented as mean ± standard deviation. The level of statistical significance was set at *P* < 0.05.

## RESULTS

The operation time and blood loss were similar in both the groups. The mean operation time for the augmented group was 63.3 ± 13.2 min, vs. 63.1 ± 17.2 min for the control group. Blood loss was 227 ± 71 cc and 242 ± 89 cc for the augmented and control groups, respectively. The duration of hospitalization was also similar for both the groups (augmented group, 4.3 ± 1.4 days; control group, 4.5 ± 1.7 days). Complications included eight superficial infections (five patients in the augmented group and three patients in the control group), one deep vein thrombosis in the augmented group and one deep infection in the control group. In the early stage, one patient in the augmented group had an intraoperative pedicular medial cortex rupture during TpBA insertion, which caused root irritation and neuralgia, necessitating revision surgery to remove the augmenter three weeks later. No other pedicle injury during TpBA insertion happened thereafter.

**Figure 2:**
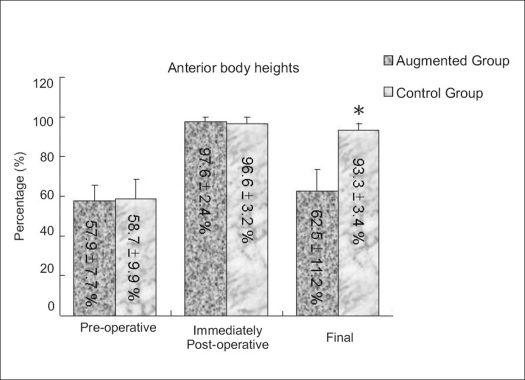
Bar diagram shows final restoration of anterior body height was better in TpBA group (93.3 ± 3.4%) in comparison to control group (62.5 ± 11.2%) (*P < 0.05)

**Figure 3:**
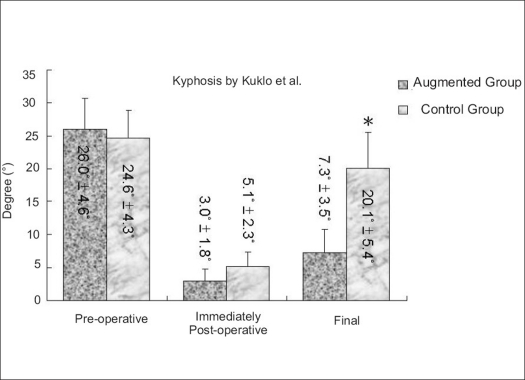
Bar diagram shows preoperative, immediate postoperative and final kyphosis in both groups. The augmented group shows better maintenance of kyphosis correction at final followup (*P < 0.05)

**Figure 4:**
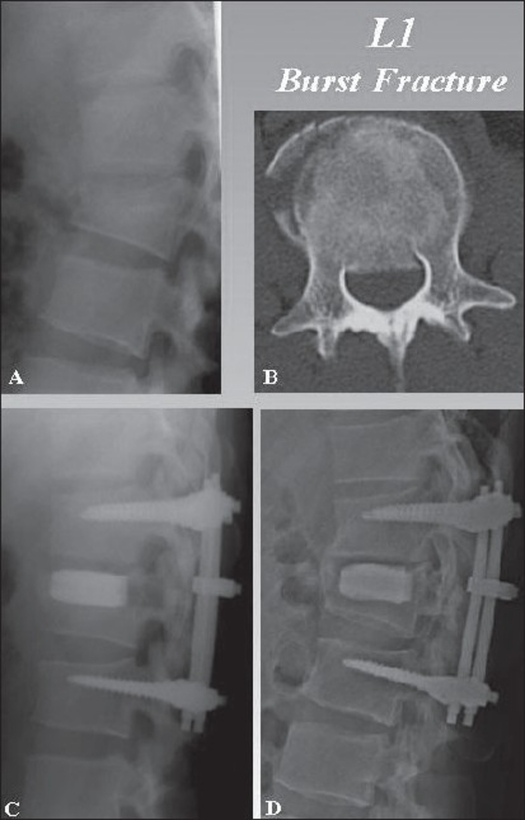
Lateral X-ray of dorsal lumbar spine (A) shows burst fracture of L1. Axial CT (B) of L1 vertebra shows burst fracture with cord compression. Lateral X-ray of immediate postoperative (C) and final followup (D) show well placed transpedicle body augmenter with restoration of anterior body height and kyphosis

Anterior vertebral height restoration and kyphotic correction was achieved and well maintained in the augmented group. The preoperative anterior vertebral height of the fractured vertebral body and kyphotic deformity were similar in both groups. The immediate postoperative anterior vertebral height and kyphosis were not different between the augmented and control groups. At the last followup, however, anterior vertebral height was greater (*P* < 0.001) in the augmented group than in the control group [Figure 2]. The reduction of kyphosis was better maintained in the augmented group [Figures 3,4].

The TpBA-augmented group had less (*P* < 0.001) implant failure (0% (n=0) vs. 23.8% (n=10) for the control group) and better clinical results. The control group had 10 implant failures and increase in kyphosis. No dislodgement of the transpedicle body augmenter occurred. Based on the pain scale of Denis *et al.*,^26^ the augmented group had more patients (*P* < 0.001) with no pain or minimal or occasional pain (Grade P1 or P2) than the control group (90.0% (n=81) vs. 66.7% (n=28)). The augmented group had fewer patients (*P* < 0.001) with severe and constant pain (Grades P4 and P5) than the control group (0% (n=0) versus 16.7% (n=7)) [Figure 4]. All patients in the augmented group and 39 patients in the control group experienced neurological recovery to Frankel Grade E. Three patients in the control group had improvement to Frankel Grade D from Frankel Grade C, but later deteriorated to Frankel Grade C because of loosening and dislodgement of the implant.

## DISCUSSION

A transpedicle body augmenter (TpBA) to reinforce the vertebral body via posterior approach has been reported to prevent implant failure and increase the clinical success rate in treating burst fracture and Kümmell's disease.^17^,^18^ Short-segment fixation alone is the most common and simple treatment for burst fractures,^4^–^6^,^8^ but it has a 20-50% incidence of implant failure and rekyphosis.^5^,^10^,^11^ This paper shows the longterm results of the TpBA function in treating thoracolumbar burst fractures.

As discussed before,^17^ there are several limitations of this study. First, there is the patient distribution problem. During the first year of learning the new technique, more patients received posterior fixation alone. In the following year, in our protocol, one patient was assigned to each group sequentially. In the last years, after patients in the augmented group showed much better results, more patients received the transpedicle body augmenter. Therefore, the patients were not randomized. To minimize bias, criteria were developed in selecting cases. Only occurrences of Magerl Type A3.3,^22^ limited to the T11-L2 region, were included. To have similar severity of burst fractures, only occurrences of a Gaines load-sharing score greater than 6 points were included, which was used as the guideline requiring the anterior approach,^23^,^24^ similar to the requirement of body reconstruction by the augmenter. Under such criteria, the patients included in our study were within a limited range. The distribution problem may be reduced partially by the patient selection. Non-blinded evaluation is another limitation. Because the TpBA is clearly evident on radiographs, blinded evaluation of treatment is impossible, although independent reviewers might have been used in evaluating the other criteria. The clinical outcomes also were determined by the treating surgeons, which could bias interpretation of findings.

Posterior short-segment fixation with the TpBA is relatively effective in preventing loss of reduction and avoiding implant failure. Loss in reduction of kyphosis after short-segment fixation (e.g. 90% by Carl *et al.*^5^ and approximately 50% by McNamara *et al.*^27^) has been numerously reported. McLain *et al*.^11^ reported that 40% of patients had greater than 10° loss of kyphosis correction. The loss of anterior body height reduction in our non-augmented group was greater compared with the 14% reported by Kramer *et al.*^8^ and 11.4% reported by Cho *et al*.^14^ The possible reason was that manual reduction by traction and compression forces^28^–^30^ and short fixation resulted in kyphosis reduction and vertebral body restoration close to the intact state. Without anterior reconstruction, the loss of restoration will be greater because of more potential space for re-collapse. Our series also showed 23.8% implant failure in the non-augmented group, which is consistent with the 20-50% failure rate reported by Benson *et al.*,^31^ McLain *et al.,*^11^ and Kuklo *et al*.^25^ Cho *et al*.^14^ reported that polymethylmethac-rylate reinforcement successfully prevented implant failure in short-segment fixation. The TpBA prevents re-collapse of vertebral body in the short term and allows longterm fracture healing without use of polymethylmethacrylate.

The longterm results showed that adjacent disc injury in thoracolumbar burst becomes a minor problem after TpBA has been used. Discs adjacent to the burst fracture are extensively damaged,^32^,^33^ and the damage is related to loss of kyphosis reduction. Transpedicle intercorporeal bone grafts^13^ were attempted to decrease postoperative rekyphosis, however, they proved ineffective because the fusion rate was only 33%.^15^ In our series, the loss of kyphosis reduction was limited in the augmented group, possibly because the internal rigid support from the augmenter restores the vertebral body and prevents failure of the posterior instrumentation, which functions to preserve the disc space and may encourage disc healing.

Once the technique is mastered, the TpBA can be used with its best benefit and without complications. To ensure the security and adequate restoration of the vertebral body, the optimal size of TpBA should be chosen. The pedicle size is different among individuals and spine levels.^34^,^35^ The pedicle to body ratio is approximately 61-69% in the T11-L2 region.^34^ With a greater pedicle to body ratio, an augmenter of optimal size can occupy and support more of the vertebral body and should be more effective in preventing body re-collapse. Because approximately 70% of burst fractures occur at the thoracolumbar junction, the augmenter can be used in the majority of burst fractures with adequate biomechanical effects. The most important precaution is not to break the pedicular medial and inferior cortex, which protect the neural component. When a bigger TpBA is needed, the pedicular superior and lateral cortex can be broken without injury of the neural tissue.

## CONCLUSION

Short-segment fixation with reinforcement of a transpedicle body augmenter is an effective and safe method to treat burst fractures. With an average followup of more than five years we showed that TpBA could ensure restoration of vertebral bodies, prevent implant failure and lead to clinical success.
